# Herbicide Options to Control Naturalised Infestations of *Cereus uruguayanus* in Rangeland Environments of Australia

**DOI:** 10.3390/plants10102227

**Published:** 2021-10-19

**Authors:** Shane Campbell, Ali Bajwa, Kelsey Hosking, Dannielle Brazier, Vincent Mellor, Melinda Perkins

**Affiliations:** 1 School of Agriculture and Food Sciences, The University of Queensland, Gatton, QLD 4343, Australia; ali.bajwa@dpi.nsw.gov.au (A.B.); vam103@maths.uq.edu.au (V.M.); melinda.perkins@daf.qld.gov.au (M.P.); 2 Tropical Weeds Research Centre, Biosecurity Queensland, Department of Agriculture and Fisheries, Charters Towers, QLD 4820, Australia; kelsey.hosking@daf.qld.gov.au (K.H.); dannielle.brazier@daf.qld.gov.au (D.B.); 3 Weed Research Unit, New South Wales Department of Primary Industries, Wagga Wagga, NSW 2650, Australia; 4 Agri-Science Queensland, Queensland Department of Agriculture and Fisheries, Cairns, QLD 4870, Australia; 5 Gatton Research Facility, Queensland Department of Agriculture and Fisheries, Gatton, QLD 4343, Australia

**Keywords:** cactus, chemical control, night-blooming cereus, Willows cactus

## Abstract

While there are many high profile Opuntioid cactus species invading rangeland environments in Australia, *Cereus uruguayanus* Ritt. ex Kiesl. has also naturalised and formed large and dense infestations at several locations. With no herbicides registered for control of *C. uruguayanus* in Australia, the primary aim of this study was to identify effective herbicides to control it using a range of techniques. This involved a large screening trial of twelve herbicides and four techniques, followed by a rate refinement trial for cut stump applications and another to test residual herbicides. Despite most treatments (except monosodium methylarsonate (MSMA)) taking a long time to kill plants, at least one effective herbicide was identified for basal bark (triclopyr/picloram), cut stump (aminopyralid/metsulfuron-methyl, glyphosate, metsulfuron-methyl, triclopyr/picloram, triclopyr/picloram/aminopyralid), stem injection (glyphosate, MSMA, triclopyr/picloram/aminopyralid) and foliar applications (aminopyralid/metsulfuron-methyl, MSMA, triclopyr, triclopyr/picloram/aminopyralid) due to their ability to kill both small and large plants. Ground application of residual herbicides was less conclusive with neither hexazinone nor tebuthiuron causing adequate mortality at the rates applied. This study has identified effective herbicides for the control of *C. uruguayanus* using several techniques, but further research is needed to refine herbicide rates and develop integrated management strategies for a range of situations and infestation sizes and densities.

## 1. Introduction

The *Cactaceae* is a large family of succulent plants that comprises more than 120 genera and 1500 species [1]. Almost all *Cacti* are native to the Americas but were introduced around the globe either deliberately or accidentally [2]. Many are cultivated for ornamental purposes, food and various industrial uses [2,3], but a large number have also become major weeds in many countries. These plants have many unique features (e.g., Crassulacean Acid Metabolism (CAM)) that allows them to establish and thrive in harsh and dry environments [2,4], making them a major threat to rangeland environments that they invade.

The majority of soil types and climatic regions in Australia are favourable for cacti growth and although the continent has no native cacti species, many exotic species have become naturalised following their deliberate introduction, mainly for ornamental purposes [3,5,6,7,8]. Naturalised cacti populations can form dense, impenetrable thickets that limit access to grazing activities and reduce habitat quality. Furthermore, the spiny stems pose a significant health risk to humans, livestock and wildlife. As a result, 27 cacti species are listed as Weeds of National Significance (WONS) in Australia, all of which belong to the Opuntioideae sub-family and the genus *Opuntia* and *Cylindropuntia* [3]. However, several *Cacti* species from other genera such as *Epiphyllum*, *Harrisia* and *Cereus* are also emerging or major weeds in different parts of the country [3,9].

**Figure 1:**
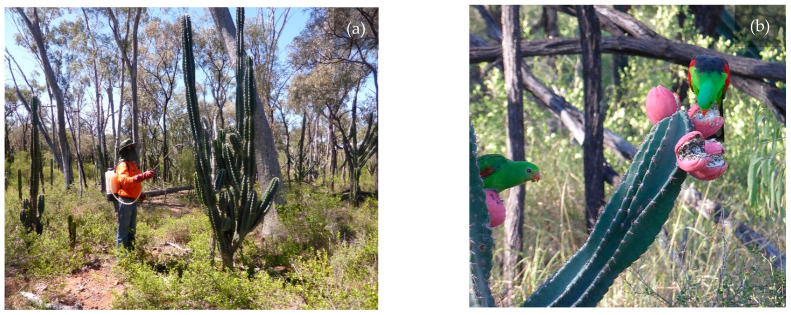
*Cereus uruguayanus* trial site showing: (**a**) foliar application of herbicide to a medium-density infestation; and (**b**) red-winged parrots (*Aprosmictus erythropterus*) feeding on *C. uruguayanus* fruit.

*Cereus uruguayanus* Ritt. ex Kiesl. of the Cactoideae sub-family is a large, columnar cactus (Figure 1a) that generally forms a spiny, multi-stemmed candelabra [9]. Its taxonomy has been confusing with it being called several other synonyms in the past, including *C. peruvianus* and *C. hildmannianus* [9,10]. Common names used in Australia include Willows cactus, apple cactus, night-blooming cereus and torch cactus. The species originates from South America and is believed to have been introduced to Australia for ornamental purposes. It is a common plant in Queensland gardens because of its interesting shape, large white flowers and edible fruits [11].

*Cereus uruguayanus* is not a WONS nor a declared weed in any state or territory of Australia, but the presence of large and multiple infestations in the Central Highlands region of Queensland has led to it being declared a priority pest species under local government legislation. Large infestations also occur in southern inland Queensland at several locations and many smaller infestations are common across southern/central Queensland [11], as well as a limited number of locations in New South Wales [12]. The species is most prevalent in mixed Eucalypt–Brigalow woodlands on light clay soils but also occurs in areas of cleared improved pasture and on a range of soil types.

Endozoochory (i.e., seed dispersal via ingestion by vertebrates) appears to be the primary dispersal mechanism of *C. uruguayanus* (Figure 1b) [11] and was attributed to the occurrence of isolated plants up to one kilometre away from a naturalised population [9]. Similarly, the invasiveness of the related cacti species *C. jamacaru* in South Africa was largely attributed to this dispersal mechanism [13]. Control options that promptly arrest flowering and fruiting are therefore desirable.

A potential *C. uruguayanus* biological control agent, Harrisia cactus mealybug *Hypogeococcus festerianus* (Hemiptera: Pseudococcidae), is already present in Australia and has been established on *C. uruguayanus* at several locations in Queensland [11]. Anecdotally, it appears to be causing some damage to *C. uruguayanus* (Craig Hunter, personal communication), whilst in South Africa it provided effective control of *C. jamacaru* [13]. However, ten years or more was required for the agent to make an observable impact on *C. jamacaru* population structure. Effective control also requires manual redistribution of the mealybug to uninfected weed populations due to its limited natural dispersal abilities [14].

Chemical control offers a more immediate solution for treating high-priority infestations, particularly those in the early stages of establishment. A range of techniques and herbicides were investigated in Australia for control of Opuntioid cacti, but not *C. uruguayanus*. As a result, there are several registered herbicides for use on Opuntioid cacti in Australia [3], but their efficacy against *C. uruguayanus* is largely unknown. In South Africa, foliar spraying of small plants or stem injection of large plants with monosodium methylarsonate (MSMA) is used for chemical control of the closely related species *C. peruvianus* [14] and *C. jamacaru* [15]. Testing is needed to determine whether MSMA also provides effective control of *C. uruguayanus*.

The primary aim of this study was to identify effective herbicides to control *C. uruguayanus* using a range of techniques. For all experiments, we tested the hypotheses that at least one of the herbicide/technique combinations used would (Alternate hypothesis) or would not (Null hypothesis) kill *C. uruguayanus* plants. An initial screening trial compared the control efficacy of MSMA with other herbicides registered for use on Opuntioid cacti in Australia. Application techniques were based on herbicide label recommendations and included basal bark and cut stump application, foliar spraying and stem injection. A second experiment investigated the efficacy of soil-applied residual herbicides, as anecdotal evidence from landholders suggested that *C. uruguayanus* was susceptible to tebuthiuron and hexazinone-based products. Promising treatments identified in the screening trial were the focus of a later, third experiment that compared cut stump application of glyphosate at varying rates with cut stump application of a triclopyr/picloram mixture.

## 2. Materials and Methods

### 2.1. Site Details

All experiments were undertaken near the township of Willows (23°44′S, 147°32′E), approximately 70 km west of Emerald, Queensland, Australia. Climatically, the area has a summer dominant rainfall (Based on Gemfields Township records) which averages 589 mm annually and ranges from 21.5 mm in July to 104.0 mm in January [16]. In terms of prevailing temperatures, monthly mean minima (Based on Emerald airport records) range between 9.1 °C (July) and 22.3 °C (January) while the monthly mean maxima range between 23.4 °C (June) and 34.6 °C (January) [17,18].

This area falls within the Brigalow Belt of Queensland and is characterised by clay soils, deep depressions (gilgais) and dense woodland vegetation dominated by the tree *Acacia harpophylla* F.Muell. ex Benth. (commonly referred to as brigalow). Experiments 1 and 2 were undertaken adjacent to each other in a remnant patch where the tree layer comprised *A. harpophylla* and several *Eucalyptus* spp., the mid-story *C. uruguayanus* and *Carissa ovata* R.Br. and the understory was sparsely covered with *Aristida* spp., and occasionally *Cenchrus ciliaris* L. Experiment 3 was undertaken nearby in a cleared area that had been planted with *C. ciliaris.* It dominated the ground layer, with isolated regrowth of *A. harpophylla* and *C. uruguayanus* scattered throughout the area.

### 2.2. Experiment 1. Screening for Suitable Herbicides and Application Techniques

The screening experiment was established in May 2016 and employed a completely randomised design with two replicate plots per treatment. Twenty-one treatments involving twelve herbicides registered for control of various cacti species in Australia and four different application methods (*viz.* basal bark, cut stump, foliar and stem injection) were selected to test their efficacy against *C. uruguayanus* (Table 1). Untreated plants served as a control treatment. In terms of modes of action (MoA), most herbicides belonged to Group 4 (Disruptors of plant cell growth; auxin mimics). The exceptions were glyphosate [Group 9: Inhibition of 5-enolpyruvyl shikimate-3 phosphate synthase (EPSP inhibition)], metsulfuron-methyl [Group 2: Inhibition of acetolactate synthase (ALS inhibitors), acetohydroxyacid synthase (AHAS)], amitrole (Group 34: Inhibition of lycopene cyclase), and MSMA (Group 0: Herbicides with unknown mode of action) [19].

Each experimental unit comprised 20 plants (spaced at least 2 m apart), approximately half of which were classified as small (<2 m in height) and the other half as large (≥2 m in height). On average, small plants were 1.03 ± 0.05 (SE) m high with an average basal diameter of 4.12 + 0.20 (SE) cm and large plants were 3.43 m ± 0.17 (SE) m high with an average basal diameter of 10.08 + 0.49 (SE) cm. As a result of the heterogeneity of the site, individual plot areas varied (*c.a.* 100–200 m^−2^) to accommodate the required number of plants in each size class. A 2 m buffer zone was maintained between the plots. 

All cut stump treatments (except aminopyralid/picloram) and basal bark treatments were applied in May 2016. Stem injection and foliar treatments were delayed until October 2016 when *C. uruguayanus* plants were in a healthier condition. At this time, we also included an aminopyralid/picloram cut stump treatment to see if this portable and easy to apply Gel (Vigilant™ 11 paste; Corteva Agriscience Australia Limited, Chatswood, NSW, Australia) had the potential for landholders to treat isolated plants when they came across them during their day-to-day work activities. 

Basal bark treatments were applied at an average operating pressure of 70 kPa using an 8 L handheld pneumatic sprayer (Swissmex^®^; Croplands Australia, Dry Creek, SA, Australia) fitted with a 0.6 m wand and an adjustable full cone nozzle. Herbicide mixture was applied to the point of run-off to the full circumference of the basal 5 cm (“thinline” treatment) or 40 cm (“traditional” treatment) of each plant stem. Cut stump treatments involved cutting off the plants ~10 cm above ground level using a battery-powered saw. Herbicide mixture was applied within ~5 s to the cut surface of the stump using the same equipment as described for the basal bark treatments. Foliar treatments involved spraying the whole plant (average operating pressure of 175 kPa) to the point of run-off using a 15 L backpack sprayer (Swissmex^®^; Croplands Australia, Dry Creek, SA, Australia) fitted with an adjustable solid cone nozzle. Stem injection treatments used a cordless drill with a 9 mm bit to insert 2–3 cm deep holes on a 45° downward angle at 10 cm intervals (hole centre to hole centre) around the circumference of each plant, at a height of ~40 cm. An NJ Phillips tree injection gun^®^ (NJ Phillips Pty. Ltd. Limited, Somersby, NSW, Australia) was then used to apply either 1 mL (amitrole/ammonium thiocyanate treatment) or 2 mL (all other treatments) of herbicide mixture into each hole.

Plants treated in May 2016 were assessed 6, 12, 17, 25, 31 and 42 months after treatment (MAT). Plants treated in October 2016 were assessed 1, 7, 13, 20, 26 and 38 MAT. At each assessment time, plant injury for basal bark, foliar and stem injection treatments was scored on a scale of 1 (alive) to 10 (dead), with each incremental increase representing a 10% increase in the proportion of dead plant material. From this, plant mortality (%) was calculated as the number of plants with an injury score of 10, expressed as a percentage of the total number of plants of that size class in the experimental unit. For cut stump treatments, separate injury scores and mortality rates were recorded for stumps and fallen stems. The same method as mentioned above was used for the fallen stems, but for the cut stump, the rating was confined to a 1 (representing alive) or a 10 (representing dead). At each assessment time, the presence/absence of flowers and fruits was also recorded.

### 2.3. Experiment 2. Evaluating Efficacy of Soil Applied Residual Herbicides

The residual herbicide experiment was initiated in November 2016 and employed a randomised complete block design comprising eight treatments and three replications. Treatments included soil application of tebuthiuron at 0.4, 0.8, 1.2 or 1.6 g a.i. m^−1^ plant height (applied as 2, 4, 6 or 8 g m^−1^ Scrubmaster^®^, 200 g kg^−1^ a.i.; FMC Crop Protection Australia, North Ryde, NSW, Australia) and hexazinone at 0.5 or 1.0 g a.i. m^−1^ plant height (applied as 2 or 4 mL m^−1^ Bobcat^®^ SL, 250 g a.i. L^−1^; Adama Australia, St Leonards, NSW, Australia). Stem injection of hexazinone was also tested in this experiment as it was not permissible within the remnant vegetation site used for Experiment 1. Untreated plants served as a control treatment.

Each experimental unit consisted of clusters of 10 plants usually located within a 200 m^−2^ area, although occasionally slightly larger areas were needed due to the variable distribution of *C. uraguayanus* at the site. On average, plants were 1.35 ± 0.04 (SE) m high with an average basal diameter of 8.55 + 0.31 (SE) cm.

For soil-applied treatments, pre-weighed tebuthiuron granules were sprinkled evenly within a 10 cm radius of the base of plants, whereas the liquid formulation of hexazinone was injected ~5 cm below ground using an NJ Phillips tree injection gun^®^ (NJ Phillips Pty. Ltd. Limited, Somersby, NSW, Australia) with a spear and brace attachment. Injections were applied within 10 cm of the base of plants and if more than one dose was required, they were evenly spaced around the base. Stem injection of hexazinone was conducted using the same technique described for Experiment 1 and resulted in a mean dosage of 0.53 ± 0.06 g a.i. m^−1^ (± s.e.). Plants were assessed 6, 11, 19, 25 and 36 MAT for plant injury score and plant mortality (as described in Experiment 1).

### 2.4. Experiment 3. Optimising Glyphosate Dose for Cut Stump Application

The cut stump experiment was initiated in November 2017 and employed a randomised complete block design, incorporating six treatments and three replications. Treatments included cut stump applications of glyphosate at rates of 45, 90, 180 or 360 g a.i. L^−1^ [Applied as 125, 250, 500 or 1000 mL L^−1^ Roundup^®^ (360 g a.i. L^−1^ present as isopropylamine salt)]. They were compared for *C. uraguayanus* control efficacy with cut stump application of a diesel-based mixture of 4 g a.i. L^−1^ triclopyr and 2 g a.i. L^−1^ picloram applied as 17 mL L^−1^ Access^®^ (240 g a.i. L^−1^ triclopyr, 120 g a.i. L^−1^ picloram). Cut stumps without herbicide application served as a control treatment. 

Each experimental unit was 20 *C. uraguayanus* plants, with half classified as small (<2 m in height) and the other half as large (≥2 m in height). On average, small plants were 0.98 ± 0.03 (SE) m high with an average basal diameter of 3.5 + 0.14 (SE) cm and large plants were 3.51 m ± 0.10 (SE) m high with an average basal diameter of 10.1 + 0.34 (SE) cm. Individual plot area averaged approximately 100 m^−2^ but varied slightly to accommodate the required number of plants in each size class. A 2 m buffer zone was maintained between the plots.

All glyphosate treatments used water as a carrier and contained 2 mL L^−1^ Pulse^®^ Penetrant (1020 g a.i. L^−1^ Polyether modified polysiloxane). Herbicides were applied using the same technique described for cut stump treatments in Experiment 1, with the exception that the cut surface of the fallen stem was also treated.

Plants were assessed 7, 13 and 25 MAT for plant injury score and plant mortality of both the stump and the fallen stem (as described in Experiment 1).

### 2.5. Statistical Analysis

All data analyses were conducted using either Minitab^®^, Version 17.3.1 (Minitab Pty Ltd., Sydney, Australia) or R and the Agricolae package [20,21]. Data expressed as percentages (i.e., mortality) were arcsine transformed prior to analysis and later back-transformed for presentation in tabular and graphic format. For each evaluation time, data from Experiment 1 and 3 were subjected to two-way analysis of variance (ANOVA) using the split-plot design (i.e., herbicide as main plot and size as sub-plot) to test whether the size of plants influenced the efficacy of the applied herbicide treatments. Data from Experiment 2 were subjected to a one-way analysis of variance for each evaluation time. With implementation times for the different techniques used in Experiment 1 split into either May or October due to the condition of plants, separate statistical analysis was undertaken based on when the treatments were applied.

Using ANOVA the significance of the sources of variation were assessed using the Fisher-Snedecor test. If the studied variable (i.e., treatments) was found to have a *p*-value lower than the significance level of α = 0.05 it was deemed to be significant, and a Fisher’s protected least significant difference (LSD) test was then used to undertake pairwise comparisons of means of all possible treatment combinations. Those combinations were identified as significantly different if their *p*-value was lower than the significance level of α = 0.05. As the two-sided LSD test *p*-value is twice that of a one-sided test, the direction can be assessed on significant results.

## 3. Results

### 3.1. Weather

Rainfall conditions during the study period varied markedly from the long-term mean (Table 2) and were above average in 2016 (+242.1 mm) and 2017 (+78.8 mm), but below average in 2018 (−228.8 mm) and 2019 (−106.2 mm) [16]. Most noticeably, the two months that preceded the implementation of the screening trial (experiment 1) in May 2016 recorded below-average rainfall. At the time plants were still considered suitable for basal bark and cut stump treatments, but foliar and stem injection treatments were delayed until plants were in a healthier condition. Fortunately, there was a wet winter period, with the months of June and July receiving more than four and six times the long-term average, respectively. This resulted in very healthy *C. uraguayanus* plants for the foliar and stem-injection treatments (implemented in October 2016) in the screening trial, as well as those in the residual herbicide trial (implemented in November 2016). The following 2016/17 wet season period (November to April) was above average largely due to a very wet January and March. The 2017 dry season was below average but was followed by an above-average wet season, which favoured the implementation of the glyphosate cut stump trial in November 2017. Overall, the remainder of the study period (May 2018 to December 2019) recorded less rainfall than the long-term mean, although some extremely high falls occurred intermittently, particularly in March and April 2019. 

### 3.2. Experiment 1. Screening for Suitable Herbicides and Application Techniques

The percentage mortality caused by different herbicides varied significantly (*p* < 0.05). Initially, plant size also had a significant influence on the efficacy of some herbicides and application techniques (*p* < 0.05) (Table 3 and Table 4).

Traditional and thinline basal bark application of triclopyr/picloram provided similar mortality (*p* > 0.05) at each assessment time (Table 3). For both treatments, plants tended to die slowly, particularly larger ones. At 12 MAT, mortality averaged 57% and 6.5% for small and large plants, respectively. By 25 MAT, 100% of all small plants had died, but mortality of larger plants was still only 71% and 85% for the traditional and thinline basal bark treatments, respectively. High mortality of large plants was not recorded until 31 MAT, when mortality averaged 95.5% across both treatments. At this time there was no significant difference (*p* > 0.05) in mortality between small and large plants. Despite the extended time taken to kill some plants, the treatments prevented reproduction, with on average 2.5% of large plants producing fruit compared to 62.5% in the untreated control.

**Figure 2:**
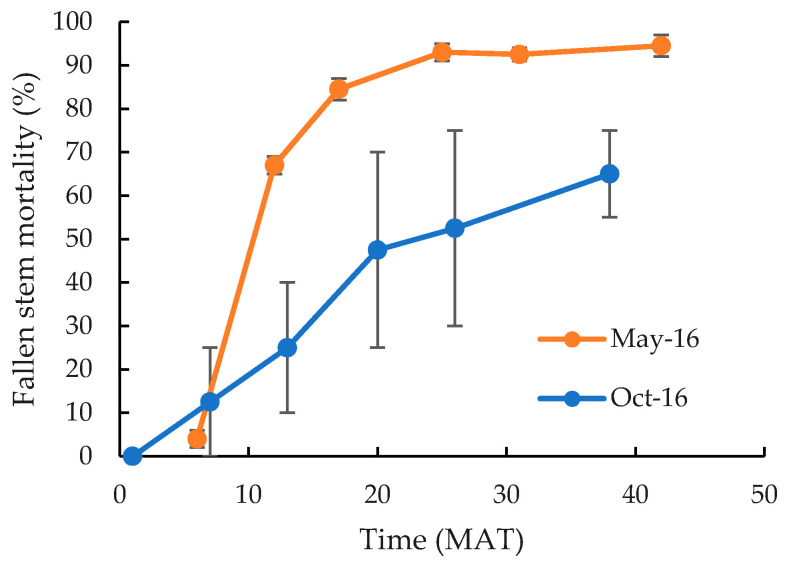
Mortality for *C. uruguayanus* fallen stems in response to cut stump herbicide application in May (orange) or October (blue) 2016 (Experiment 1). Vertical bars represent the standard error of the mean.

There were no significant differences (*p* > 0.05) between cut stump treatments implemented in May 2016, with all causing high mortality of both the cut stump (Table 3) and the fallen stem section (Figure 2), irrespective of the size of plants. However, whilst 100% mortality of the cut stump was recorded 6 MAT it took much longer for the fallen stems to dry out and die (Figure 2). At 6 MAT, only 4% of fallen stems were dead. This increased almost linearly to 85% 17 MAT, before slowly increasing to a maximum death rate of 93% 25 MAT, with minimal changes occurring thereafter. The cut stump application of aminopyralid/picloram in October 2016 also resulted in 100% mortality of the cut stump (Table 3), but as for the May applications, fallen stems took longer to die. However, even then a greater proportion of fallen stems survived for longer, with only 63% recorded as dead 38 MAT (Figure 2). Those that were still alive had re-attached to the ground and developed new roots and in some instances new stems. Nevertheless, reproduction was minimal with fruits recorded on less than 1% of plants across all cut stump treatments.

Foliar spraying resulted in highly significant differences (*p* < 0.05) between herbicide treatments at all monitoring times (Table 4). There were also significant (*p* < 0.05) herbicide × size class interactions 1 and 7 MAT, but the effect of size class was not significant (*p* > 0.05) thereafter. MSMA was the fastest-acting foliar herbicide, with small plants most susceptible initially (Table 4). Within a couple of days, blistering of the stem was a notable treatment effect and by 1 MAT 86% and 21% of small and large plants were dead, respectively. In contrast, all other treatments recorded nil mortality 1 MAT, except triclopyr that has a slight effect on small plants (2% mortality). 

At 7 MAT, most of the foliar-applied herbicides were having a significantly greater (*p* < 0.05) effect on small plants than large plants, with the exception of those containing metsulfuron-methyl which was causing low mortality across both size classes (0–18%). Although slower to act (particularly on larger plants) than MSMA, both formulations of triclopyr/picloram/aminopyralid and triclopyr ended up causing 100% mortality of both size classes by 20 MAT and were not significantly different (*p* > 0.05) to MSMA (99% mortality). Aminopyralid/metsulfuron-methyl and picloram + fluroxypyr were even slower to act but reasonably effective, averaging 82% and 73% mortality 26 MAT, respectively. Amitrole/ammonium thiocyanate and metsulfuron-methyl were both ineffective, particularly metsulfuron-methyl (5% mortality 38 MAT) which was not significantly different (*p* > 0.05) to the control at all monitoring times (Table 4). Furthermore, a proportion of both small and large plants recorded fruit production following treatment with amitrole/ammonium thiocyanate (6 and 37%) and metsulfuron-methyl (24 and 63%), with the latter not significantly different (*p* > 0.05) to the untreated control (27 and 62.5%). In contrast, all other foliar treatments recorded nil fruit production during the 38-month monitoring period.

For stem injection treatments, highly significant herbicide × size class interactions (*p* < 0.05) occurred at both 1 and 7 MAT, but thereafter only herbicide treatments exhibited significant differences (*p* < 0.05). At 1 and 7 MAT, all herbicide treatments caused higher mortality of small plants compared to large plants, except triclopyr/picloram/aminopyralid at 1 MAT (average of 0%) and MSMA (≥96%) at 7 MAT. As for foliar applications, MSMA was again the fastest acting herbicide, causing even higher mortality early on compared to if it was foliar sprayed (Table 4). After one month, 100% of small and 42% of large plants were dead, and by 13 MAT all remaining large plants had died. Although slower to act (particularly on larger plants), glyphosate caused 99% mortality across both size classes 20 MAT. MSMA and glyphosate also prevented fruit production following application. Amitrole/ammonium thiocyanate and triclopyr/picloram/aminopyralid were the slowest acting herbicides, but eventually (38 MAT) caused high mortality (96–99%) of both small and large plants (Table 4). In the interim, a small proportion of plants (≤6%) continued to produce fruits.

### 3.3. Experiment 2. Evaluating Efficacy of Soil Applied Residual Herbicides

**Figure 3:**
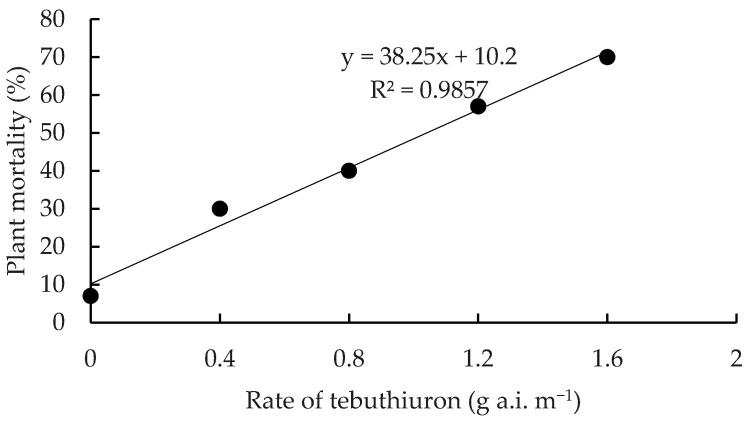
The relationship between application rate of tebuthiuron and mortality (%) of *C. uruguayanus* plants.

Significant differences in mortality occurred between residual herbicide treatments (*p* < 0.05) at all monitoring times. For soil-applied herbicide treatments, most mortality occurred over a 19-month period (Table 5). The exception was the higher rates of tebuthiuron where mortality increased up to 25 and 36 MAT at rates of 1.2 and 1.6 g a.i. m^−1^ of plant height, respectively. After 36 MAT, a clear linear rate response was evident with maximum mortality (70%) recorded at the highest applied rate of 1.6 g a.i. m^−1^ of plant height (Figure 3). Both rates of soil-applied hexazinone were ineffective, with mortality averaging only 23.5% at 36 MAT. In contrast, stem injection of hexazinone was highly effective and faster acting with 90% mortality recorded 11 MAT (Table 5). 

### 3.4. Experiment 3. Optimising Glyphosate Dose for Cut Stump Application

At all monitoring times, a significant interaction (*p* < 0.05) occurred between herbicide treatments and size classes for mortality of both the stump and fallen stem of *C. uruguayanus* plants (Table 6). Plants cut off but not treated (controls) with herbicide, exhibited low mortality of the cut stump initially but over time it increased steadily. By 25 MAT, mortality of the cut stump portion of control plants was significantly higher in large plants compared to small plants, averaging 58% and 31%, respectively. Death of the fallen stem section also increased over time at a rate that was not significantly different (*p* > 0.05) for small and large plants. At 7, 13 and 25 MAT, mortality averaged 33.5%, 38.5% and 71.5%, respectively. 

Of the herbicide treatments, at 7 MAT triclopyr/picloram had caused significantly higher mortality (≥95%) of cut stumps than the three lowest rates of glyphosate, but not the highest rate (i.e., 360 g a.i. L^−1^) which averaged 91% mortality across both size classes. At this time, only the lowest rate of glyphosate recorded a differential size class response with small and large plants averaging 84 and 35% mortality, respectively. Small plants continued to be more susceptible at this lower rate 13 MAT but not at 25 MAT. 

Over time, mortality increased and by 25 MAT all herbicide treatments recorded ≥97% and 90% of cut stumps for small and large plants, respectively. Triclopyr/picloram and the highest concentration of glyphosate killed all cut stumps of large plants, whereas the lowest concentration of glyphosate killed 90%.

Death of fallen stems of both size classes increased over time, but for small plants, it was almost always significantly higher (*p* < 0.05) in herbicide treatments (except triclopyr/picloram at 7 MAT) compared to untreated fallen stems in the control treatment (Table 6). At the final monitoring (25 MAT), 100% of fallen stems from small plants were dead if treated with herbicide, whilst 29% of untreated stems remained alive. In contrast, spraying the cut section of fallen stems of large plants did not significantly increase mortality, averaging 85% across all treatments (including the control).

## 4. Discussion

While there is a broad range of herbicides recommended for the control of invasive cactus species in Australia, using various techniques [3], this study found that a smaller number were effective on *C. uruguayanus*. Even those effective were often slow to cause high mortality (>90%) of *C. uruguayanus*, particularly on larger plants which tended to take longer to die than smaller ones. Nevertheless, at least one herbicide demonstrated high efficacy using basal bark (triclopyr/picloram), cut stump (aminopyralid/metsulfuron-methyl, glyphosate, metsulfuron-methyl, triclopyr/picloram, triclopyr/picloram/aminopyralid), stem injection (glyphosate, MSMA, triclopyr/picloram/aminopyralid) and foliar techniques (aminopyralid/metsulfuron-methyl, MSMA, triclopyr, triclopyr/picloram/aminopyralid) due to their ability to kill both small and large plants. Ground application of residual herbicides was less conclusive and warrants further investigation.

Basal barking was one of the slowest acting techniques particularly on larger plants, which recorded minimal mortality (<20%) 17 MAT. However, using triclopyr/picloram high mortality (≥90%) was eventually achieved 25 and 31 MAT, for small and large plants respectively. This slow activity is partly due to the way the herbicide affected the plant. We observed that necrosis occurred initially where the herbicide was directly applied (i.e., bottom 40 cm of the stem). The remaining upper portion of the plant would then slowly die over time while still upright, although the stems of several larger plants fell over and died lying on the ground. Differences in response of small and large woody plants to basal bark treatments are not uncommon, with smaller plants generally more susceptible [22,23,24]. 

Both the traditional and thinline basal bark methods are used to control invasive woody weeds [22,23,24,25,26]. In the current study, they provided similar efficacy and either could be used depending on the preference of the operator. *Leucaena leucocephala* (Lam.) de Wit is another species that was also found to be equally susceptible to both basal bark techniques [25].

Compared to basal barking, cut stump applications were much quicker to cause high mortality of treated stumps for all herbicides that were tested. This is consistent with what was achieved for a range of woody weeds using this technique, provided plants are cut off close to ground level and the herbicide is applied immediately onto the cut surface to allow rapid absorption [26], as occurred in the current study. Experiment 3 demonstrated that the application of herbicide onto the cut stump is essential for high mortality. In the absence of herbicide, mortality of the cut stumps averaged only 31% and 58% for small and large plants, respectively. Even lower mortality was reported for woody weeds such as *Ligustrum sinense* Lour. (14% mortality) [27] and *Ailanthus altissima* (Mill.) Swingle (21% mortality) [28] if herbicide was not applied to the cut stump. In the current study, the height that plants were cut off was *c.a* 10 cm aboveground which caused high mortality after herbicide application. At greater cut heights, plant mortality could be expected to decline as was reported for several woody weeds [26,29]. For example, mortality of *Calotropis procera* (Aiton) W.T. Aiton after application of 2,4-D butyl ester averaged 67% when plants were cut 5 cm above the ground and 0% when the plants were cut 20 cm above ground level [26].

While the stumps of plants treated using the cut stump method died relatively quickly, the fallen stem sections took much longer to die. In some instances, fallen stems reattached to the ground and new stems shot along the length of the original stem. This was a rare occurrence following cut stump applications in May 2016, but more common when undertaken in October 2016. This may be associated with the duration of dry conditions following treatment, with prolonged dry periods potentially facilitating higher mortality. Plants treated in May were exposed to the whole dry season period (although it was wetter than average), while those treated in October experienced a much shorter dry period before the start of the wet season which typically commences by November in sub-tropical/tropical environments. Spraying of the cut section of fallen stems to increase mortality proved successful for small plants but not larger ones. Despite the limited regrowth from fallen stem sections, landholder experience associated with other tall-growing opuntia cactus often makes them hesitant to undertake cut stump applications. A big difference however is that we observed that *C. uruguayanus* maintains its structure when it falls onto the ground, whereas cladodes and stem sections readily break off on many other cactus species. It is these cladodes/sections that can result in the formation of a new plant [3].

Foliar spraying demonstrated the greatest variability of all the techniques tested. Across the eight foliar herbicide treatments, the rate at which plants died and overall mortality varied markedly. Some herbicides were ineffective on small and large plants, others only controlled small plants, while a limited few caused high mortality irrespective of the size of plants. MSMA was the fastest-acting herbicide and killed *C. uruguayanus* plants more quickly than all other treatments. It is used for the control of *C. peruvianus* [30] and *C. jamacaru* [31] in South Africa and is particularly effective on smaller plants. It is also recommended for several Opuntioid cacti in Australia, but due to its poison status, strict safety guidelines are prescribed to minimise risks to the operator. In the current study, three slower-acting herbicides eventually caused very high mortality of both small and large *C. uruguayanus* plants. They were two formulations of triclopyr/picloram/aminopyralid (Grazon™ Extra and Tordon™ RegrowthMaster) and triclopyr. They are all considered less hazardous than MSMA and are commonly used for spraying a range of other invasive woody weeds and several other cactus species [3]. While they were capable of causing high mortality of large plants, from an application perspective this technique would be most suitable for treating smaller plants (i.e., ≤2 m) with larger plants controlled using other options, such as stem injection.

Stem injection was highly effective at killing both small and large plants of *C. uruguayanus*, with glyphosate performing as well as MSMA despite being slower acting. These herbicides are recommended for control of other cactus species [3,30] using the stem injection technique given their succulent nature and the ability of systemic herbicides to translocate rapidly. Minimisation of damage to non-target species and low costs are also considered advantages of the stem injection technique for cactus control [30,31]. The broad spectrum triclopyr/picloram/aminopyralid (Tordon™ RegrowthMaster) also proved effective and would be an appropriate option in areas where there are multiple woody weeds growing amongst *C. uruguayanus*. Hexazinone was also highly effective when stem injected, but its use would be restricted to more open areas to minimise non-target impacts.

Ground application of two residual herbicides failed to cause high mortality of *C. uruguayanus*, but further investigation is warranted. While hexazinone performed poorly, tebuthiuron demonstrated a linear rate response, so testing higher rates may result in higher mortality of *C. uruguayanus* and should be explored. Furthermore, no non-target damage was observed in the stem injection treatment, but despite being injected into the soil dead *C. ciliaris* tussocks were observed around all ground applied hexazinone treatments. It was particularly evident on the lower slope of treated plants where in some instances dead *C. ciliaris* plants were up to 3 m away.

Whilst this study has identified several effective herbicides and application techniques for the control of *C. uruguayanus*, further research is warranted. In particular, future studies should focus on identifying the most cost-effective control options for infestations of differing sizes and densities. This should include exploration of other potential techniques (i.e., mechanical, biological) as some herbicides can cause adverse effects on the environment and they may not be applicable in all situations, such as environmentally sensitive areas. This is particularly pertinent for residual herbicides such as tebuthiuron and hexazinone that can remain in the soil for extended periods [32,33], but even herbicides that are thought to be relatively safe to use (e.g., glyphosate) could be problematic in some situations [34]. Irrespective, a single technique is rarely effective for control of a particular weed and an integrated approach is usually needed to deal with not only the original plants but also the subsequent regrowth that could continue to appear whilst there is a residual seed bank, or if the site continues to be re-infested from external sources (such as a nearby infestation) [35]. An enhanced understanding of the ecology and population dynamics of *C. uruguayanus* would also be advantageous and provide insights into how this plant is likely to respond to imposed treatments over time.

## 5. Conclusions

While still in the early stages of invasion in Australia, at several locations *C. uruguayanus* has formed large and dense infestations in rangeland environments. With no herbicides registered to control *C. uruguayanus*, this study focused on screening a range of chemicals and application techniques. At least one effective herbicide was identified for basal bark, cut stump, stem injection and foliar applications, but the ground application of residual herbicides was ineffective at the rates applied. Based on these findings, a minor use permit has now been approved in Queensland (Australia) for landholders to use several herbicides and techniques to control *C. uruguayanus* [11]. Landholders need to be patient though and allow sufficient time (at least two years) before confirming the effectiveness of control programs, as this weed is often slow to react to herbicide applications. Further research is also needed, particularly to refine rates for some of the more effective herbicides and to identify integrated management strategies for a range of situations (i.e., different infestation sizes and densities) and control both the initial infestation and subsequent regrowth.

## Figures and Tables

**Table 1:** Application techniques and herbicides screened for *C. uruguayanus* control efficacy in Experiment 1.

Technique	Herbicide Active Ingredient(s)	Mode of Action (Group)	Herbicide Trade Name	Rate (g a.i. L^−1^)	Carrier	Treatment Date
Basal bark (traditional)	Triclopyr (240 g L^−1^)/picloram (120 g L^−1^)	4	Access™	4/2	Diesel	May 2016
Basal bark (thinline)	Triclopyr (240 g L^−1^)/picloram (120 g L^−1^)	4	Access™	24/12	Diesel	May 2016
Cut stump	Aminopyralid (375 g kg^−1^)/metsulfuron-methyl (300 g kg^−1^)	4/2	Stinger™	1.5/1.2	Water ^1^	May 2016
Cut stump	Aminopyralid (4.47 g L^−1^)/picloram (44.7 g L^−1^)	4	Vigilant™ II	4.47/44.7	- ^2^	Oct 2016
Cut stump	Glyphosate (360 g L^−1^)	9	Roundup^®^	360	- ^2^	May 2016
Cut stump	Metsulfuron-methyl (600 g kg^−1^)	2	Brush-Off^®^	1.2	Water ^1^	May 2016
Cut stump	Triclopyr (240 g L^−1^)/picloram (120 g L^−1^)	4	Access™	4/2	Diesel	May 2016
Cut stump	Triclopyr (200 g L^−1^)/picloram (100 g L^−1^)/aminopyralid (25 g L^−1^)	4	Tordon™ RegrowthMaster	10/5/1.25	Water ^1^	May 2016
Foliar	Aminopyralid (375 g kg^−1^)/metsulfuron-methyl (300 g kg^−1^)	4/2	Stinger™	0.15/0.12	Water ^1^	Oct 2016
Foliar	Amitrole (250 g L^−1^)/ammonium thiocyanate (220 g L^−1^)	34	Amitrole T	10/8.8	Water ^1^	Oct 2016
Foliar	Metsulfuron-methyl (600 g kg^−1^)	2	Brush-Off^®^	0.12	Water ^1^	Oct 2016
Foliar	MSMA (720 g L^−1^)	0	Daconate 720^®^	18	Water ^1^	Oct 2016
Foliar	Picloram (240 g L^−1^) + fluroxypyr (333 g L^−1^)	4	Stuka Flexi + Starane™ Advanced	0.6/0.5	Water ^1^	Oct 2016
Foliar	Triclopyr (600 g L^−1^)	4	Garlon™ 600	18	Water ^1^	Oct 2016
Foliar	Triclopyr (300 g L^−1^)/picloram (100 g L^−1^)/aminopyralid (8 g L^−1^)	4	Grazon™ Extra	1.5/0.5/0.04	Water ^1^	Oct 2016
Foliar	Triclopyr (200 g L^−1^)/picloram (100 g L^−1^)/aminopyralid (25 g L^−1^)	4	Tordon™ RegrowthMaster	5/2.5/0.625	Water ^1^	Oct 2016
Stem injection	Amitrole (250 g L^−1^)/ammonium thiocyanate (220 g L^−1^)	34	Amitrole T	250/220	- ^2^	Oct 2016
Stem injection	Glyphosate (360 g L^−1^)	9	Roundup^®^	360	- ^2^	Oct 2016
Stem injection	MSMA (720 g L^−1^)	0	Daconate 720^®^	240	Water	Oct 2016
Stem injection	Triclopyr (200 g L^−1^)/picloram (100 g L^−1^)/aminopyralid (25 g L^−1^)	4	Tordon™ RegrowthMaster	40/20/5	Water	Oct 2016
Control	none		-	-	-	-

^1^ Mixture also contained 2 mL L^−1^ Pulse Penetrant (1020 g a.i. L^−1^ Polyether modified polysiloxane) (Nufarm Australia, Laverton North, Vic.). ^2^ Herbicide applied neat and not diluted.

**Table 2:** Monthly and annual precipitation at the Willows township from 2016–2019 and the long-term mean for each month and year.

Year	Jan.	Feb.	Mar.	Apr.	May	June	July	Aug.	Sept.	Oct.	Nov.	Dec.	Tot.
2016	161.0	183.8	26.4	3.8	0.0	116.2	132.0	22.2	93.0	11.4	43.3	38.0	831.1
2017	233.4	3.0	117.6	0.0	3.0	2.8	22.6	5.8	0.0	94.4	109.6	75.6	667.8
2018	33.0	107.6	31.0	4.8	4.0	5.4	1.0	0.0	0.0	126.6	20.2	26.6	360.2
2019	2.2	0.0	163.4	212.2	0.0	15.2	17.4	12.0	0.0	28.8	23.0	8.6	482.8
Mean	104.0	91.7	56.0	33.7	36.4	24.5	21.5	24.2	26	40.2	57.9	84.1	589.0

**Table 3:** Mortality of small (S) and large (L) *C. uruguayanus* plants 6, 12, 17, 25, 31 and 42 months after treatment (MAT) in response to herbicide treatments applied in May 2016 (Experiment 1).

Herbicide Treatment	Plant Mortality (%) ^1^									
	6 MAT		12 MAT		17 MAT		25 MAT		31 MAT ^2^	42 MAT ^2^
	S	L	S	L	S	L	S	L		
Control ^3^	0b	0b	0d	0d	0d	0d	0d	0d	2b	5b
Basal bark										
Triclopyr/picloram (Traditional)	4b	0b	58b	6c	71b	19c	100a	71c	96a	98a
Triclopyr/picloram (Thinline)	5b	0b	56b	7c	80b	17c	100a	85b	95a	100a
Cut stump ^4^										
Aminopyralid/metsulfuron-methyl	100a	100a	100a	100a	100a	100a	100a	100a	100a	100a
Glyphosate	100a	100a	100a	100a	100a	100a	100a	100a	100a	100a
Metsulfuron-methyl	100a	96a	100a	100a	100a	100a	100a	100a	100a	100a
Triclopyr/picloram	100a	100a	100a	100a	100a	100a	100a	100a	100a	100a
Triclopyr/picloram/aminopyralid	100a	100a	100a	100a	100a	100a	100a	100a	100a	100a

^1^ Means within an assessment time that do not share a letter are significantly different (*p* < 0.05) according to Fisher’s LSD Test. ^2^ For 31 and 42 MAT measurements, plant size data were combined as they were not significantly different (*p* > 0.05). ^3^ Untreated plants served as a control treatment. ^4^ Plant mortality relates only to the cut stump. Mortality of fallen stems was assessed separately and the results presented in Figure 2.

**Table 4:** Mortality of small (S) and large (L) *C. uruguayanus* plants 1, 7, 13, 20, 26 and 38 months after treatment (MAT) in response to herbicide treatments applied in October 2016 (Experiment 1).

Herbicide Treatment	Plant Mortality (%) ^1^							
	1 MAT		7 MAT		13 MAT ^2^	20 MAT ^2^	26 MAT ^2^	38 MAT ^2^
	**S**	**L**	**S**	**L**				
Control ^3^	0f	0f	0i	0i	0f	0e	0.5d	2e
Foliar								
Aminopyralid/metsulfuron-methyl	0f	0f	11g–i	18f–h	42d	74b	82b	93bc
Amitrole/ammonium thiocyanate	0f	0f	25d–h	0i	17e	32d	38c	40d
Metsulfuron-methyl	0f	0f	0i	0i	0f	0e	0.5d	5e
MSMA	86b	21d	100a	84bc	99ab	99a	99a	99ab
Picloram + fluroxypyr (tank mix)	0f	0f	29d–g	0i	31de	51cd	73b	83c
Triclopyr	2ef	0f	90ab	58cd	99ab	100a	100a	100a
Triclopyr/picloram/aminopyralid ^4^	0f	0f	53c–e	10g–i	76c	100a	100a	100a
Tricloypr/picloram/aminopyralid ^5^	0f	0f	47d–f	22e–h	88c	100a	100a	100a
Stem injection								
Amitrole/ammonium thiocyanate	6e	0f	44d–f	0i	38de	66bc	83b	96a–c
Glyphosate	35cd	0f	95ab	28d–g	91bc	99a	100a	100a
MSMA	100a	42c	100a	96ab	100a	100a	100a	100a
Tricloypr/picloram/aminopyralid ^5^	0f	0f	45d–f	3h–i	22de	79b	99a	99ab
Cut stump ^6^								
Aminopyralid/picloram	0f	0f	100a	100a	100a	100a	100a	100a

^1^ Means within an assessment time that do not share a letter are significantly different (*p* < 0.05) according to Fisher’s LSD Test. ^2^ For 13, 20, 26 and 38 MAT measurements, plant size data were combined as they were not significantly different (*p* > 0.05). ^3^ Untreated plants served as a control treatment. ^4^ Grazon™ Extra. ^5^ Tordon™ RegrowthMaster. ^6^ Plant mortality relates only to the cut stump. Mortality of fallen stems was assessed separately and the results presented in Figure 2.

**Table 5:** *Cereus uruguayanus* plant mortality 6, 11, 19, 25 and 36 months after treatment (MAT) in response to soil-applied residual herbicides in Experiment 2.

Treatment	Plant Mortality (%) ^1^				
	6 MAT	11 MAT	19 MAT	25 MAT	36 MAT
Control ^2^	0c	0e	0e	3e	7d
Tebuthiuron					
0.4 g a.i. m^−1^	20a–c	27b–d	30b–d	30b–e	30cd
0.8 g a.i. m^−1^	20ab	27bc	40b–d	40b–d	40bc
1.2 g a.i. m^−1^	17a–c	23b–d	47bc	57bc	57bc
1.6 g a.i. m^−1^	24ab	47b	60b	63b	70ab
					
Hexazinone					
0.5 g a.i. m^−1^	0c	3de	10de	20de	27cd
1 g a.i. m^−1^	7bc	10c–e	20cd	20c–e	20cd
Stem injected ^3^	45a	90a	93a	93a	93a

^1^ Means within a column that do not share a letter are significantly different (*p* < 0.05) according to Fisher’s LSD test. ^2^ Untreated plants served as a control treatment. ^3^ Stem injection of hexazinone was included as a comparator.

**Table 6:** Mortality (%) of the cut stump and fallen stem from small (S) and large (L) *C. uruguayanus* plants 7, 13 and 25 months after treatment (MAT) in response to cut stump and herbicide treatments applied in Experiment 3.

Herbicide Treatment	Cut Stump Mortality (%) ^1^						Fallen Stem Mortality (%) ^1^					
	7 MAT		13 MAT		25 MAT		7 MAT		13 MAT		25 MAT	
	S	L	S	L	S	L	S	L	S	L	S	L
Control	4g	14fg	17c	27c	31d	58c	44c–e	23e	50e–g	27g	71b	72b
Triclopyr/picloram	100a	95ab	100a	100a	100a	100a	76a–c	20e	82b–d	49e–g	100a	93ab
Glyphosate												
45 g a.i. L^−1^	84b–d	35ef	87a	60b	97ab	90b	79ab	37de	88a–c	44fg	100a	92ab
90 g a.i. L^−1^	77cd	60de	95a	87a	97ab	95ab	82ab	32e	94a	50e–g	100a	81ab
180 g a.i. L^−1^	88bc	69cd	100a	93a	100a	97ab	63b–d	59b–d	85a–d	71c–e	100a	86ab
360 g a.i. L^−1^	97ab	85b–d	100a	96a	100a	100a	91a	35de	94ab	62d–f	100a	88ab

^1^ Means within an assessment time that do not share a letter are significantly different (*p* < 0.05) according to Fisher’s LSD test.

## Data Availability

Not applicable.
